# Integrating imaging-based classification and transcriptomics for quality assessment of human oocytes according to their reproductive efficiency

**DOI:** 10.1007/s10815-023-02911-y

**Published:** 2023-08-23

**Authors:** Xavier Viñals Gonzalez, Christopher Thrasivoulou, Roy Pascal Naja, Srividya Seshadri, Paul Serhal, Sioban Sen Gupta

**Affiliations:** 1 Preimplantation Genetics Group, Institute for Women’s Health, University College London, 84-86 Chenies Mews, Bloomsbury, London, WC1E 6HU UK; 2 Research Department of Cell and Developmental Biology, University College London, Rockefeller Building, London, WC1E 6DE UK; 3 The Centre for Reproductive and Genetic Health, 230-232 Great Portland St, Fitzrovia, W1W 5QS London UK

**Keywords:** Human oocyte, Quality, Outcome prediction, Transcriptome, Image analysis

## Abstract

**Purpose:**

Utilising non-invasive imaging parameters to assess human oocyte fertilisation, development and implantation; and their influence on transcriptomic profiles.

**Methods:**

A ranking tool was designed using imaging data from 957 metaphase II stage oocytes retrieved from 102 patients undergoing ART. Hoffman modulation contrast microscopy was conducted with an Olympus IX53 microscope. Images were acquired prior to ICSI and processed using ImageJ for optical density and grey-level co-occurrence matrices texture analysis. Single-cell RNA sequencing of twenty-three mature oocytes classified according to their competence was performed.

**Result(s):**

Overall fertilisation, blastulation and implantation rates were 73.0%, 62.6% and 50.8%, respectively. Three different algorithms were produced using binary logistic regression methods based on “optimal” quartiles, resulting in an accuracy of prediction of 76.6%, 67% and 80.7% for fertilisation, blastulation and implantation. Optical density, gradient, inverse difference moment (homogeneity) and entropy (structural complexity) were the parameters with highest predictive properties. The ranking tool showed high sensitivity (68.9–90.8%) but with limited specificity (26.5–62.5%) for outcome prediction. Furthermore, five differentially expressed genes were identified when comparing “good” versus “poor” competent oocytes.

**Conclusion(s):**

Imaging properties can be used as a tool to assess differences in the ooplasm and predict laboratory and clinical outcomes. Transcriptomic analysis suggested that oocytes with lower competence may have compromised cell cycle either by non-reparable DNA damage or insufficient ooplasmic maturation. Further development of algorithms based on image parameters is encouraged, with an increased balanced cohort and validated prospectively in multicentric studies.

**Supplementary Information:**

The online version contains supplementary material available at 10.1007/s10815-023-02911-y.

In the era of assisted reproduction techniques (ART), implantation is one of the areas with the greatest interest since failures in this process are the most frequent cause of an unsuccessful treatment [1]. It is the current consensus that compromised embryo quality, rather than impaired endometrium function is one of the main causes of age-related fertility decline [2]. Moreover, understanding oocyte quality is imperative not only to maximise treatment outcomes but also to provide a comprehensive assessment of the fertility status of the female patient. With the aid of polarised microscopy systems, new literature of mature metaphase II (MII) stage has become available. Observation of the meiotic spindle in MII oocytes made possible a first insight in assessing nuclear maturity in human oocytes [3]. Ultimately, oocyte competence is acquired when the meiotic cycle is resumed and cytoplasmic elements organise and re-distribute along the ooplasm [4]. To date, the optimal markers for cytoplasmic competence are still to be defined [5].

Artificial intelligence (AI) has gained momentum as a tool in the field of fertility. Deep learning algorithms applied to biological images from embryos are hoped to establish a universal system to grade and select pre-implantation embryos. Khosravi et al. [6] used 50,392 time lapse images from 10,148 human embryos to develop the “STORK” framework, which was compared with the classification given by different embryologists [6]. The algorithm developed showed an accuracy of 96.94% when identifying good- and poor-quality blastocysts based on pregnancy probabilities, which “outperforms” the embryologist criteria according to the authors. Chavez-Badiola et al. [7] analysed a total of 1231 blastocyst images and their computing tool “ERICA” (embryo ranking intelligent classification algorithm) showed a 79.0% positive predictive value with an accuracy of 70% for predicting euploidy [7].

Used broadly in medical imaging, texture analysis is an effective approach to increase the data obtainable from images. The specific distribution of subcellular components endows cells of texture [8]. Texture parameters are a mathematical descriptions of image characteristics as a function of the spatial changes in neighbour pixel intensities. For more than a decade, the relevance of such analysis has proven to be key to characterise subtle properties of biologic samples mainly based on magnetic resonance images [9, 10]. Castellano et al. [16] established some principles for texture analysis for medical images using grey-level co-occurrence matrices (GLCM), although the application of such analysis to understand human oocyte quality has not been sufficiently explored in literature. With the aid of image analysis, differences in texture in the early stages of the first cell cycle have been reported when comparing MII oocytes to in vitro matured MII oocytes [11]. Authors reported differences in texture parameters post-injection time, polar body extrusion, pronuclei appearance and pronuclei fading but not immediately before cytokinesis. Authors suggested that the differences observed in texture parameters could be attributable to an inadequate cytoplasmic maturation.

While texture parameters give information related to concepts such as homogeneity, structural complexity, uniformity and level of order within an image, optical density (OD) describes the propagation of light through a sample and its value depends on processes of absorption, reflection or scattering of the light as a result of the sample’s composition. The present study aims to understand the potential of imaging and texture parameters, based on modulation contrast microscopy, to predict oocyte competence in terms of fertilisation, embryo development and implantation. To provide a novel method to assess human oocyte quality, an imaging ranking algorithm based on regression models (ranking tool) is proposed. Looking at the oocyte’s transcriptome, it has been established that the maturation stage is the main factor defining internal composition with almost 6000 genes differentially expressed between GV and MII stages [12]. The same group identified around 450 genes uniquely expressed in single stages. Although the presence of some transcripts may naturally happen due to the progression and arrest of the cell cycle, others are susceptible to external factors such as female age, obesity and/or medical conditions (i.e. endometriosis) [13, 14]. Moreover, using a single-cell approach, transcriptomic profiles from good- and poor-quality oocytes according to their imaging ranking scores were compared.

## Materials and methods

This retrospective study was performed at private fertility clinic (London, UK) in collaboration with the EGA Institute for Women’s Health (University College London, UK). Ethical approval was granted from the London – Surrey Research Ethics Committee (reference: 18/LO/1849; Go project ID: 253968). The project is also registered on a publicly accessible database (clinicaltrials.gov ID: NCT03683290).

### Study cohort

The study included oocytes from patients undergoing intracytoplasmic sperm injection (ICSI) using own fresh oocytes following a short antagonist protocol. For the purpose of the present study, the following exclusion criteria were applied: female age > 39 years, suboptimal response (< 4 metaphase II oocytes retrieved), BMI over 30 kg/m^2^, uterine pathology, endometriosis, polycystic ovarian syndrome, recurrent pregnancy loss. Oocyte donation and severe masculine factor (< 5 M/ejaculate) were also excluded. A total of 1126 cumulus-oocyte-complex (COCs) were retrieved, resulting in 957 MII oocytes suitable for ICSI, derived from 102 patients who had fertility treatment from May 2019 to July 2020. After insemination, a total of 699 resulted in correct fertilisation (two pronuclei, PN/two polar bodies, PB) and 438 developed to the blastocyst stage. Known implantation data of 114 embryos was available from fresh single blastocyst replacements. Implantation was defined by the observation of foetal heart on ultrasound scan after 7 weeks of positive pregnancy test.

### Oocyte retrieval and denudation

Oocyte retrieval was performed at ~ 37 h post trigger injection. The embryologist examined the aspirated fluid in the search of COCs using an integrated stereomicroscope placed in an IVF chamber (HD Scientific, Australia) maintained at 37.5 °C and 6% CO_2_. The COCs were then moved to pre-warmed and gassed dishes filled with fertilisation media (ORIGIO® Sequential Fert™, human serum albumin (HSA) 5 mg/ml). Removal of cumulus cells was performed at 39–41 h post trigger injection using recombinant human hyaluronidase (Cumulase, CooperSurgical®). Oocytes were then scored for maturity based on the presence/absence of polar bodies or germinal vesicles in a round dish containing micro drops of HEPES buffer supplemented with HSA and overlaid with paraffin oil.

### Embryo culture and transfer

ICSI was performed ~ 40–42 h post trigger injection. After injection, oocytes were individually placed in different wells in an EmbryoSlide (Vitrolife®) containing ORIGIO® SAGE 1-Step™ (CooperSurgical®) overlaid with paraffin oil. Embryoscope culture was uninterrupted for 5/6 days and conditions were 37 °C and low oxygen conditions (5%-oxygen, 6%-carbon dioxide and 89%-nitrogen). Embryos were graded in accordance with modified Gardner and Cornell’s group scoring system in preparation for embryo transfer [15]. The best embryo was selected and transferred to the female patient using ultrasound guidance.

### Experimental design for oocyte cytoplasmic analysis

#### Image acquisition

**Fig. 1:**
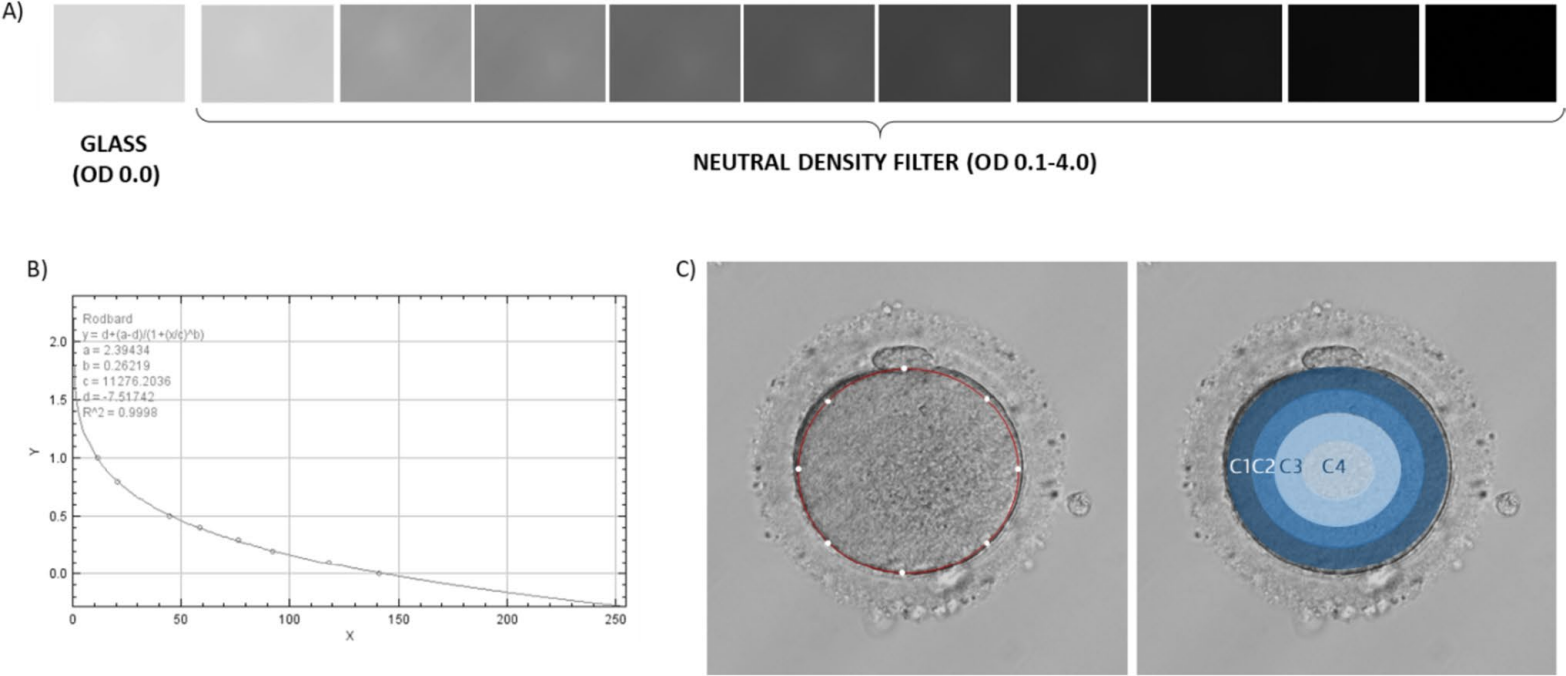
Image acquisition and analysis. **A** For each image acquisition session, a series of images must be taken using a calibrated optical density step filter slide. The variable neutral density filter used had 10 steps with a density range of 0.1–4.0 OD. An image of a clear glass was also taken to account for the background. **B** Calibration curve obtained using ImageJ when allocating OD values to the measured grey values from the calibration images. **C** Once the system was calibrated, the region of interest (oocyte cytoplasm) was selected and OD together with texture parameters were measured. Four concentric circles were drawn to analyse OD in the different areas (C1–C4)

Light microscopy was conducted with an Olympus IX53 microscope (Zeiss, Germany) including a Nikon D300 camera with a C mount (Nikon, USA). Illumination conditions were stable throughout image acquisition. For each image acquisition session, a series of images were taken using a calibrated OD step filter slide (Fig. 1A). The variable neutral density filter used had 10 steps with a density range of 0.1–4.0 OD (NDL-25S-4, Thorlabs Ltd.). Clear glass measurements were performed to establish 0.0 OD. At a medium magnification (× 20), Kohler illumination alignment was performed where the field diaphragm was closed, the specimen (oocyte) centred and the focus directed on the oolemma (central/middle focal plane). The condenser and diaphragm were corrected to achieve optimum focus while ensuring polarisation filter and intensity of light was adjusted to the optimum conditions for image visualisation. Images were acquired before ICSI injection.

#### Image parameters

Image analysis was done using ImageJ software (National Institutes of Health). Prior to analysis of an image, the software was calibrated to ensure reproducibility and comparable results amongst all images taken at different times. In order to generate a calibration/standard curve, images obtained using the variable neutral density filter were uploaded and assigned with their corresponding OD value (Fig. 1B). Once the calibration curve was generated, oocyte images were uploaded and analysed. Using an area selection tool, the cytoplasm of the sample was selected, and OD was measured (Fig. 1C). To see if there was any spatial differentiation of OD within the oocyte, four concentric circles were drawn to define four areas or “rings” (C1–C4; 1/4 radius apart) for an OD distribution analysis. The ring area was calculated using the formula:

$$OD\; Ring =\frac{Integrated\; density\; (Outer\; cirle-Inner\; circle)}{Area\; (Outer\; circle-Inner\; circle)}$$

The OD difference between C1–C2 and C3–C4 was calculated and expressed as a percentage, variable “gradient” (GRAD). Texture analysis was carried out using GLCM as described in literature [16]. The GLCM parameters in this study were computed on 8-bit images for four orientations: 0°, 90°, 180° and 270°. For each orientation, three measurements were performed considering a distance step of 1, 2 and 3 pixels. The average value was calculated for each parameter. GLCM parameters were studied to get information regarding image homogeneity (angular second moment, ASM; inverse difference moment, IDM), heterogeneity (contrast, CON), smoothness (correlation, CORR) and randomness (entropy, ENT).

#### Set-up experiment 1: repeatability of the measurement

For fifty metaphase II oocytes from the cohort, two images were taken in the same orientation, and OD measurement was done. For both measurements, the microscope and optic system were prepared each time to account for the setting up methodology. There was no operator selection based on morphology for the oocytes included in the cohort. A Pearson correlation coefficient was obtained when comparing both measurements. A Wilcoxon signed-rank test was used to compare the two sets of measurements from the same oocytes.

#### Set-up experiment 2: accuracy of the focus

Ten MII-stage oocytes from the cohort were analysed for OD at different focal planes, considering the equatorial plane when the oolema was in focus. Five focal planes, under and above, were considered. The distance between focal planes was 15 µm. A Pearson correlation analysis was performed to compare the average OD from the 10 focal planes with the OD from the equatorial plane.

#### Set-up experiment 3: angle and distance dependency of GLCM parameters

Ten MII-stage oocytes from the cohort were analysed for GLCM parameters at different angles (0°, 90°, 180° and 270°), considering ten-pixel distances to observe potential angle-dependent properties and step differences. Kruskal–Wallis *H* test was used to determine differences in GLCM parameters considering the different orientations for each of the distances.

### Statistical analysis

Calculations were performed using the Statistical Package for Social Sciences version 23 (SPSS Inc., Chicago, USA). Statistical significance was fixed at a 5% level (*p* = 0.05). Normal distribution for continuous variables was tested using Shapiro–Wilk test while equality of variances was assessed using the Brown–Forsythe’s test. Mann–Whitney *U* test was used to determine the differences in imaging parameters between independent groups (fertilised/not fertilised; blastulation/failed blastulation; implanted/not implanted).

Exact values of the imaging parameters (continuous) were re-coded in categories considering their quartiles (Q1–Q4) for all injected oocytes. Fertilisation (Fert), blastulation (Blast) and implantation (Impl) percentages for each quartile were calculated and the two quartiles with highest percentage for each of the outcomes defined the “optimal range” for each of the variables. A binary variable (in/out of the optimal range) was created and used for binary logistic regression analysis. Receiver operating characteristic (ROC) curves were used to test the predictive value of imaging variables included in the model and the different outcomes. Common statistically significant predictive parameters were then considered for the elaboration of a decision tree algorithm based on a best-fit model from the results of the regression analysis, as previously described in literature [17]. For each predictive model accuracy, positive predictive value (PPV), sensitivity and specificity were calculated to understand its predictive properties.

### Transcriptomic analysis

For single-oocyte RNA-seq analysis, 50 oocytes were collected from 28 female patients meeting the aforementioned inclusion criteria. A total of 46 samples were sequenced successfully from which 14 of them were GV-stage oocytes, nine MI-stage oocytes and 23 MII-stage oocytes. A total of fourteen MII oocytes were classified with good competence scores (ranking tool scores: 1 and 2) whereas nine were classified with poor competence scores (ranking tool scores: 3 and 4). Samples were prepared with the NEBNext Single Cell/Low Input RNA Library Prep Kit for Illumina (New England BioLabs®, USA, #E6420). The protocol was followed as published, with the only modification being a switch of the NEBNext Adaptors for IDT xGen UDI-UMI adaptors. Samples were sequenced on the NextSeq 500 with a v2.5 High Output 75 cycle sequencing kit (20,024,906). Samples were demultiplexed and FASTQ generated with Illumina’s bclConvert software (v3.7.5). Quality control of the reads and bioinformatic analysis was performed using Galaxy platform [18]. Limma-voom tool [19] was used for identifying differentially expressed genes according to the different sample information provided to the software. Detectable fold change (*FC* = 5.61) for the comparison was calculated as previously described by Hart et al. [20] at 0.9 power and 0.01 FDR (*p*-value). In order to identify which pathways include the differentially expressed genes, gene ontology (GO) analysis was performed using the GoSeq tool [21].

## Results

The study analysed 957 MII oocytes, derived from 102 patients ranging 25–39 years of age (35.3 ± 2.6, mean ± standard deviation). Patient ovarian reserve test parameters were anti-Mullerian hormone (AMH) 18.1 ± 11.58 pmol/l (95% CI 17.1–19.1), antral follicle count (AFC) 20.5 ± 11.1 (95% CI 19.5–21.4) and body mass index (BMI) 23.3 ± 3.3 (95% CI 23.1–23.6). Reason of infertility was: 72 patients (70.6%) were diagnosed with primary infertility whilst the rest (30, 29.4%) were diagnosed with secondary infertility out of which 13 (43.3%) had a previous live birth, 9 (30%) had one to two miscarriages (< 10 weeks), 7 (23.3%) had a pregnancy termination for social reasons and one (3.4%) had a previous ectopic pregnancy. In our cohort, average male age was 37.9 ± 5.9 and the sperm parameters were: volume 2.7 ± 1.16 ml (95% CI 2.68–2.85), total sperm count 61.8 ± 49.28 (95% CI 58.13–65.5) M/ejaculate and total motile sperm 13.5 ± 8.6 M/ejaculate (95% CI 12.87–14.17). Overall maturation, fertilisation and blastulation (utilisation) rates were 957/1126 (84.9%), 699/957 (73.0%) and 438/699 (62.6%), respectively. A total of 114 single blastocyst replacements yielded an implantation rate of 50.8% (58/114).

### Imaging system reproducibility, focus and orientation

**Fig. 2:**
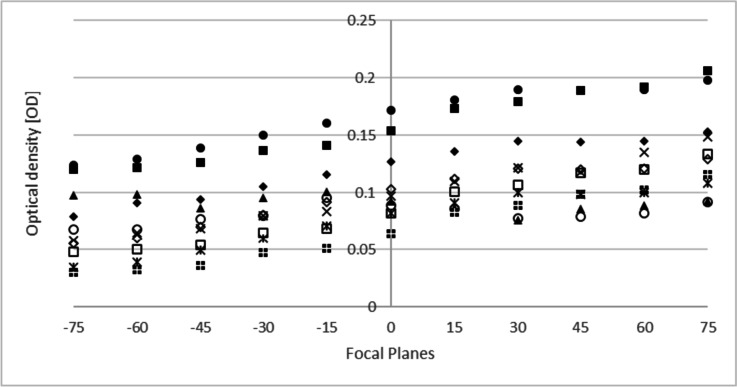
Focal plane and OD measurement. A total of ten focal planes, above and under the equatorial plane, were studied for ten MII-stage oocytes. The distance between focal planes was 15 µm. Each marker type represents an oocyte, focal plane “0” represents the equatorial plane

Repeated OD measurements for fifty MII oocytes resulted in very similar results (Supplementary Fig. 1). The coefficient of determination amongst both measurements was high (*r*^2^ = 0.9843) and statistically significant (*p* < 0.001). A Wilcoxon signed-rank test showed that the second measurement did not translate into statistically significant change in OD (*z* =  − 0.7112, *p* = 0.477). Figure 2 shows the variation of OD measurements in the different focal planes analysed for ten MII oocytes. The arithmetic mean of the different focal planes was compared against the OD value from the equatorial plane. The coefficient of determination amongst both values was high (*r*^2^ = 0.984) and statistically significant (*p* < 0.001). Hence, the equatorial plane is therefore a good representation of the different focal planes within the volume of the oocyte.

GLCM texture parameters showed similar values along the different angles studied (Supplementary Fig. 2). No angle-dependent property was observed as there were no statistically significant differences amongst the values for each parameter considering the different angle of orientation (*p* > 0.05). ASM, CORR and IDM values decreased as distance of step increased, while CON and ENT show the opposite. Three step distances (1–3) were selected to account for local variations of GLCM parameters, as variation of such decreases with an increased step.

### Imaging selection parameters based on regression analysis

#### Fertilisation

A total of 699 (73%) oocytes resulted in correct fertilisation while the rest which did not fertilise were: 129 (13.5%) 0PN, 24 (2.5%) 1PN, 25 (2.6%) 3PN and 80 (8.4%) degenerated post-injection. Table 1 summarises the imaging and texture parameters analysed for oocytes forming zygotes (*n* = 699) from those which did not (*n* = 258). The median values were statistically different for texture variables and OD gradient but not for OD only. The quartiles for each parameter are presented in Table 4 with their corresponding fertilisation rate. The quartiles with highest fertilisation rates were defined into “optimal” range for the subsequent analysis. Binomial logistic regression analysis was performed to quantify the effect of optimal ranges. The model identified OD, OR_OD_ 4.71 (95% CI 3.396–6.535) followed by GRAD, OR_GRAD_ 2.31 (95% CI 1.466–2.771) and IDM, OR_IDM_ 2.01 (95% CI 0.425–1.064) as the variables with most predictive value characterising fertilising oocytes. The predictive properties of this model were: 0.767 (AUC, 95% CI 0.734–0.801), accuracy of 76.6% and PPV of 79.9%. Sensitivity and specificity were 88.6% and 37.7%.

**Table 1:**
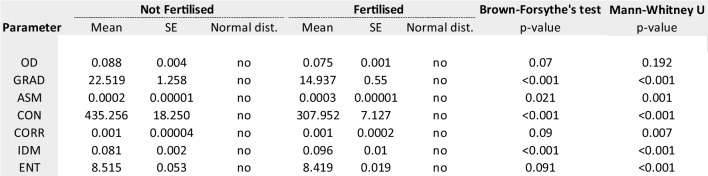
Exact values for imaging parameters of oocytes resulting in correct fertilisation (*n* = 699) vs those failing to fertilise (*n* = 258)

#### Blastulation

A total of 645 embryos were cultured up to 6 days of embryo development and 438 (67.9%) developed to the blastocyst stage while the rest (32.1%) did not. Table 2 summarises the imaging and texture parameters analysed for oocytes forming blastocysts from those which did not. The median values were statistically different for IDM, ENT and OD gradient. The quartiles for each parameter are presented in Table 4 with their corresponding blastulation rate. Binomial logistic regression analysis identified OD, OR_OD_ 2.756 (95% CI 1.905–3.988) followed by GRAD, OR_GRAD_ 2.686 (95% CI 1.878–3.841), IDM OR_IDM_ 0.572 (95% CI 0.335–0.979) and ENT, OR_ENT_ 0.330 (95% CI 0.201–0.540) as the variables with the highest predictive value characterising oocytes developing to blastocysts. The predictive properties of this model were: 0.722 (AUC, 95% CI 0.691–0.763), accuracy of 67.9% and 73.5% PPV. Sensitivity and specificity were 90.9% and 30.7%.

**Table 2:**
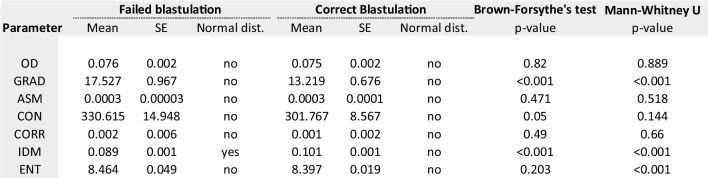
Exact values for imaging parameters of oocytes resulting in correct blastulation (*n* = 438) vs those failing to form blastocyst (*n* = 207)

#### Implantation

A total of 114 single embryo transfers were performed and 58 (50.9%) resulted in an ongoing pregnancy (implanted embryo) confirmed via ultrasound scan after ~ 7 weeks of a positive pregnancy test (mean female age, 34.3 ± 3.51). Table 3 summarises the imaging and texture parameters analysed for oocytes leading to an implanting blastocyst from those which did not. The median values were statistically different for CON, ENT and OD gradient. The quartiles for each parameter are presented in Table 4 with their corresponding implantation rate. Binomial logistic regression analysis identified OD, OR_OD_ 7.756 (95% CI 2.122–28.356) followed by GRAD, OR_GRAD_ 0.087 (95% CI 0.014–0.528) and ENT, OR_ENT_ 0.019 (95% CI 0.004–0.094) as the variables with the highest predictive value characterising oocytes leading to implanting blastocysts. The predictive properties of this model were: 0.891 (AUC, 95% CI 0.883–0.949), accuracy of 80.7% and 80% PPV. Sensitivity and specificity were 92.8% and 65.8%.

**Table 3:**
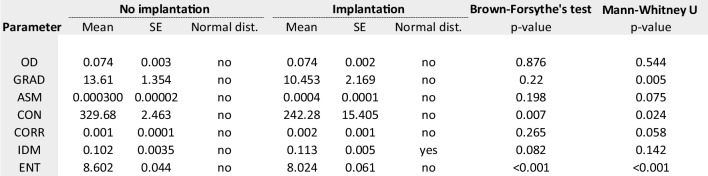
Exact values for imaging parameters of oocytes resulting in clinical pregnancy (implantation, *n* = 58) vs those which not (*n* = 56)

**Table 4:**
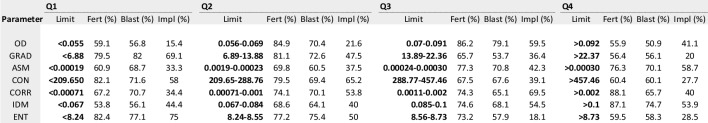
Exact values of the imaging parameters grouped in quartiles (Q1, Q2, Q3 and Q4) from 957 inseminated (mature) oocytes. Fertilisation (Fert), blastulation (Blast) and implantation (Impl) percentages for each quartile are presented. The two quartiles with highest percentage for each of the outcomes defined the “optimal range” for each of the variables

### Oocyte scoring system based on an imaging comprehensive algorithm for reproductive efficiency ranking tool

OD and GRAD have shown to be the common parameters with predictive value for fertilisation, blastulation and implantation. Hence, a two-tier algorithm based on both parameters is suggested allowing the system to score oocytes in four categories. The scoring system has an accuracy and positive predictive value of 73.1%/78.9%, 70.2%/72.3% and 65.7%/65.5% for fertilisation, blastulation and implantation. Specificity and sensitivity were 42.6%/84.4%, 26.5%/90.8% and 62.5%/68.9% for fertilisation, blastulation and implantation. The ranking tool algorithm classified 294 oocytes (30.7%) as competency score 1, 231 (24.1%) score 2, 209 (21.8%) score 3 and the rest (223, 23.3%) as score 4.

### Correlation between transcriptomic landscape and oocyte scoring system based on imaging parameters

**Fig. 3:**
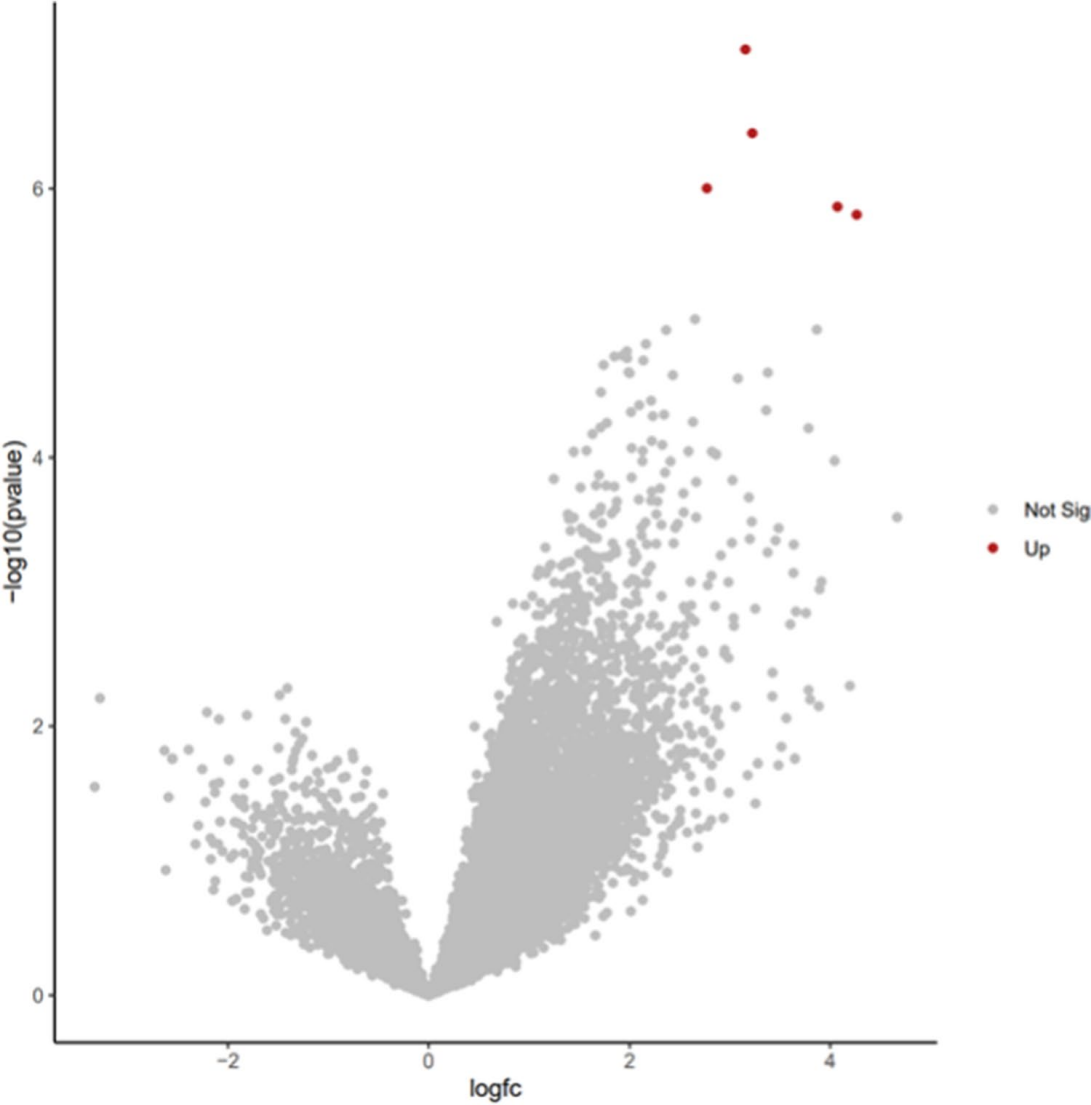
Differentially expressed genes according to imaging classification. Volcano plot illustrating the relationship between gene FDR *p*-values (< 0.01) and fold changes amongst the samples. The vertical and horizontal bars represent the filtering settings

**Fig. 4:**
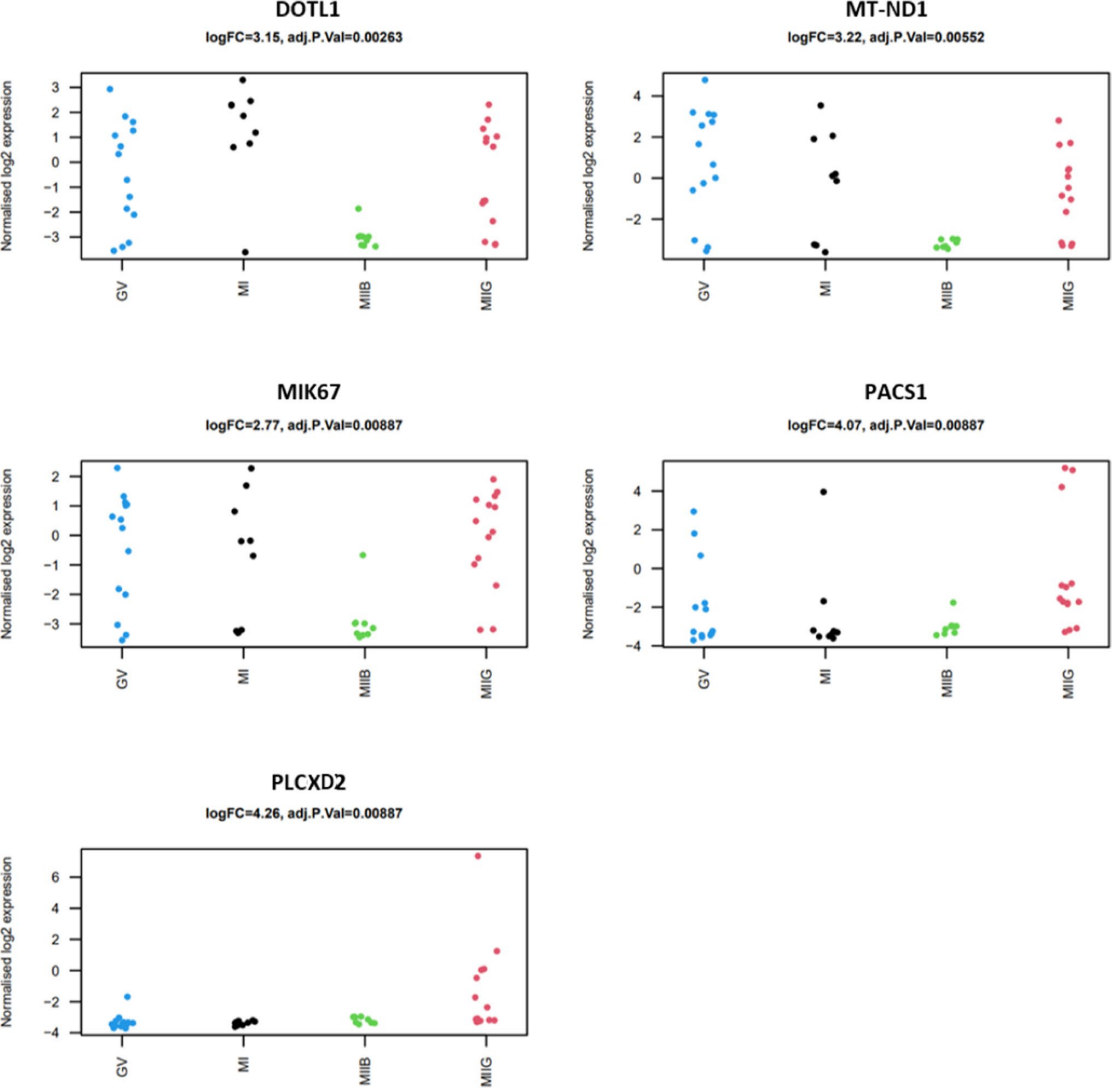
Strip charts of differentially expressed genes between high competent (MIIG) and low competent (MIIB) oocytes. The expression of the differentially expressed genes has also been illustrated together with their relative expression in immature GV and MI stages of development

A total of five DEGs were identified when comparing “good” versus “poor” competent oocytes (all upregulated), after filtering for logFC 2.48 and FDRp-value < 0.01 (Fig. 3). The upregulated genes in highly competent oocytes were *MT-ND1* (mitochondrially encoded NADH:ubiquinone oxidoreductase core subunit 1), *PACS1* (phosphofurin acidic cluster sorting protein 1), *DOT1L* (DOT1 like histone lysine methyltransferase), *PLCXD2* (phosphatidylinositol specific phospholipase C X domain containing 2) and *MIK67* (marker of proliferation Ki-67). These genes have found to be related to metabolism of RNA, cellular metabolism and cell cycle. Figure 4 illustrates logFC expression for these genes in the immature (GV/MI-stage) and “high” competent (MIIG) and “low” competent (MIIB) mature oocytes.

## Discussion

With the aid of image analysis, the present study aimed to correlate oocyte imaging properties such as optical density and texture parameters with laboratory and clinical outcomes. Seven parameters (OD, GRAD, ASM, CON, CORR, IDM, ENT) were analysed in a cohort of 957 metaphase-II oocytes. Three different algorithms were produced using binary logistic regression methods based on “optimal” quartiles. For fertilisation prediction, three variables (OD, GRAD and IDM) were the most promising yielding a model with 0.767 AUC, 76.6% accuracy and 79.9% PPV (88.6% sensitivity, 37.7% specificity). Blastulation was better predicted when ENT was also added to the variables relevant for fertilisation prediction. The algorithm predicted blastulation with 0.722 AUC, 67.9% accuracy and 73.5% PPV (90.9% sensitivity, 30.7% specificity). The final algorithm with implantation as the outcome variable identified three variables (OD, GRAD and ENT) with the most prominent prediction capabilities. The model had an AUC of 0.891, accuracy of 80.7% and positive predictive value of 80% (92.8% sensitivity, 65.8% specificity). A ranking tool based on OD and GRAD allowed classification of oocytes in four different scores (1–4). Oocytes with score “1” showed the highest competence in terms of fertilisation, blastulation and implantation. The ranking tool showed high sensitivity (68.9–90.8%) but with limited specificity (26.5–62.5%) for outcome prediction.

The application of texture analysis to understand human oocyte quality has not been sufficiently explored in literature. IDM describes image local homogeneity and it has higher values when all elements in the image are similar (homogeneous). IDM has been identified by our models as a promising covariable to predict fertilisation and blastulation, where higher values for IDM increase the odds for fertilisation and blastulation competence. Compared to the ranking tool, IDM increases accuracy and PPV for fertilisation and blastulation. Together with IDM, ENT enhances the predictive capability of the model for blastulation only. ENT measures the randomness of an image and its value is higher in complex textures. Given our results, ENT shows to be lower in oocytes developing to the blastocyst stage with higher implantation potential. When comparing the independent models with the ranking tool scoring system, texture parameters reduce the false positive rates and improve specificity for blastulation and implantation.

AI tools based on blastocyst images have been able to predict implantation with an accuracy ranging from 64.3 to 97.5% [6, 22, 23]. Blastocysts models have been largely based on time lapse technology images, which provide a standardised and robust methodology for data acquisition. In our approach, images were taken prior to ICSI as a representation of a basal status using an inverted microscope and Hoffman modulation contrast technique. Our methodology was reproducible and the analysis of the equatorial focal plan showed to be a good representation of the different focal planes within the volume of the oocyte. Oocyte texture imaging analysis using time lapse technology has been attempted before [11], but the potential effect of injection and basal status was not determined.

### Transcriptomic landscape and oocyte competence

Transcriptomic analysis revealed that a total of five differentially expressed genes were identified when comparing “good” versus “poor” competent oocytes. All of them showed to be upregulated in “good” oocytes and were related to transcription, metabolism and cell cycle. Upregulation of PLCXD2 in competent oocytes could indicate that the cell is ready to support transduction events upon fertilisation. PLCXD2 is a phosphatidylinositol-specific phospholipase C subtype which is thought to be able to increase the turnover of IP from an unknown phospholipid pool [24]. The ability of regulate cytosolic calcium translates in an indirect ability to maintain RNA concentration and increase protein synthesis as shown in other cell types [25]. Furthermore, not significant upregulation of other genes (such as AFF2) hints the capability to control processes by which mRNA is rearranged in different proteins. Analysis of normalised FC expression for PLCXD2 in both GV and MI suggests that this gene is downregulated in immature oocytes.

*MT-ND1* gene encodes for some of the proteins integrating this complex, which is upregulated in mature oocytes with high potential based on the imaging ranking tool scores. Although not significant, three genes encoding for the cytochrome C (*MT-CO1*, *MT-CO2* and *MT-CO3*) and cytochrome b (*MT-CYB*, associated with complex III) show also to be upregulated in such oocytes. Hence, upregulation of genes related to the respiratory chain in high competent oocytes could be a positive indicator of correct metabolism, required for spindle maintenance and correct functioning of the cell. Interestingly, Lan et al. [26] analysed mitochondrial gene expression in human cumulus cells and observed no difference in relative expression of MT-ND1 when comparing cumulus from GV, MI and MII; although there was a negative trend noted when comparing immature from mature cumulus cells [26]. In our data, a negative trend was also observed when comparing normalised FC expression of *MT-ND1* in GV, MI and competent MII oocytes. Interesting non-competent MII oocytes classified by the imaging ranking tool showed complete downregulation of these, suggesting that metabolic processes may be disrupted.

As part of gene regulation requirements, DNA has the ability to modify its configuration by process of acetylation and deacetylation (addition/removal of acetyl groups to histone-lysine residues). Relevant to this process, *PACS1* gene codes for a protein (PACS1) which has the ability to promote stabilisation of histone deacetylases, necessary for DNA damage repair and genomic stability. PACS1 knockdown mouse models result in proteasome-mediated degradation of deacetylases, compromised chromatin maturity and increase replication stress-induced DNA damage [27]. Additionally, mouse models PACS1 downregulation could impair kinetochore function in oocytes leading to defective microtubule attachments [28]. Hence, upregulation of *PACS1* in high competent oocytes could be a positive indicator of chromatin health, spindle integrity and reduced susceptibility to DNA damage. Interestingly, some immature oocytes display upregulation of PACS1, suggesting that such features may be acquired from early maturation stages. Also related to DNA damage response, DOT1L encodes for a histone methyltransferase which is dispensable for the early steps of mouse development [29]. Changes in DNA methylation status have a direct impact in transcription regulation (suppressed by methylation) which in turn plays a role in cell cycle regulation. Silencing of DOT1L in mouse GV oocytes resulted in metaphase block during meiosis, hence highlighting its function in meiosis progression in mouse models which could be similar in human as found here to be upregulated in highly competent oocytes according to the imaging ranking tool [30]. DOT1L normalised expression in our samples showed similar expression in GV, MI and competent MII oocytes, majorly upregulated. Interestingly, none of the non-competent MII oocytes showed upregulation for this gene.

Upon nuclear envelope disassembly, MIK67 protein maintains individual mitotic chromosomes dispersed in the cytoplasm while preventing to adopt single chromatin mass structure [31, 32]. Depletion of this protein in mouse models results in asymmetric distribution of nucleolar components [33] as chromosome associations result in impaired spindle assembly and metaphase plate formation [31]. In a mouse model, Winking et al. [34] showed that pKI-67 is likely to be maternally transmitted as there was little to no detection of this protein in sperm cells [34]. This is also supported by our results as similar expression pattern was observed for GV, MI and competent MII oocytes (majorly upregulated). Mature oocytes with lower competence showed consistent downregulation for this gene.

Although the non-invasive character of the approach, the oocytes require to be isolated from their cumulus cells for ooplasm correct visualisation. Therefore, oocytes inseminated via conventional IVF could not be assessed with our approach. To minimise bias and the effect of sperm parameters, oocytes injected with poor sperm characteristics were excluded. Still, the effect of the male gamete was not quantified in the present study. Our results support the notion that the competence to fertilise, develop and implant comes partly, but not solely, from the female gamete [35]. Further development of the algorithms based on image analysis is encouraged, with an increased balanced cohort and validated prospectively in multicentric studies. Moreover, the use of AI algorithms could certainly increase the number of features available to study. There are different clinical scenarios where an oocyte scoring system would be beneficial, such as oocyte vitrification for fertility preservation, prediction of oocyte competence on oocyte donation programs and as a feedback tool to plan future treatment cycles for patients using their own eggs. Establishing developmental potential from an early stage is key in modern reproductive science. To date, from a clinical perspective, oocyte competence has mainly been based on efficiency/success of a treatment cycle. While nuclear maturity can be visually predicted, current knowledge does not provide a comprehensive model of the ooplasm optical properties along the maturation process of the female reproductive cell. Currently, there is no consensus on the ideal marker(s) of oocyte competence and this subject should be further investigated.

### Supplementary Information

Below is the link to the electronic supplementary material.

Supplementary file1 (DOCX 107 KB)
