# Supplementary material for: Integrating imaging-based classification and transcriptomics for quality assessment of human oocytes according to their reproductive efficiency
Source: J Assist Reprod Genet. 2023 Aug 23;40(11):2545–56. doi: 10.1007/s10815-023-02911-y (PMC10643756; doi:10.1007/s10815-023-02911-y)
Supplement: Supplementary file 1 — Supplementary file1 (DOCX 107 KB) [file 10815_2023_2911_MOESM1_ESM.docx]

**SUPPLEMENTAL MATERIAL**

**Supplementary Figure 1**  Comparison of OD values of fifty different MII-stage oocytes measured two times, with a complete set up step in between

**
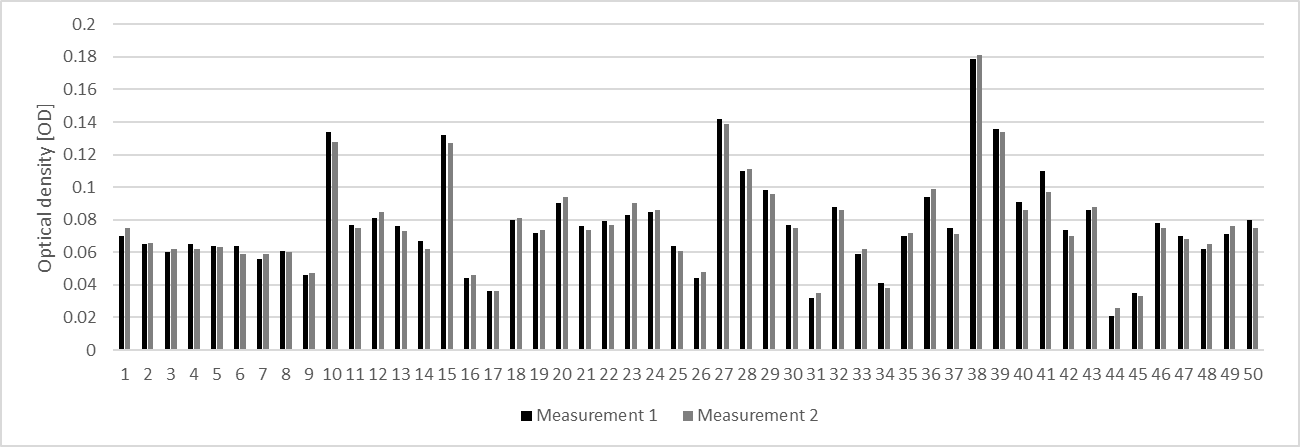
**

**Supplementary Figure 2** GLCM parameters as a function of distance considering different orientations for ten MII-stage oocytes

**
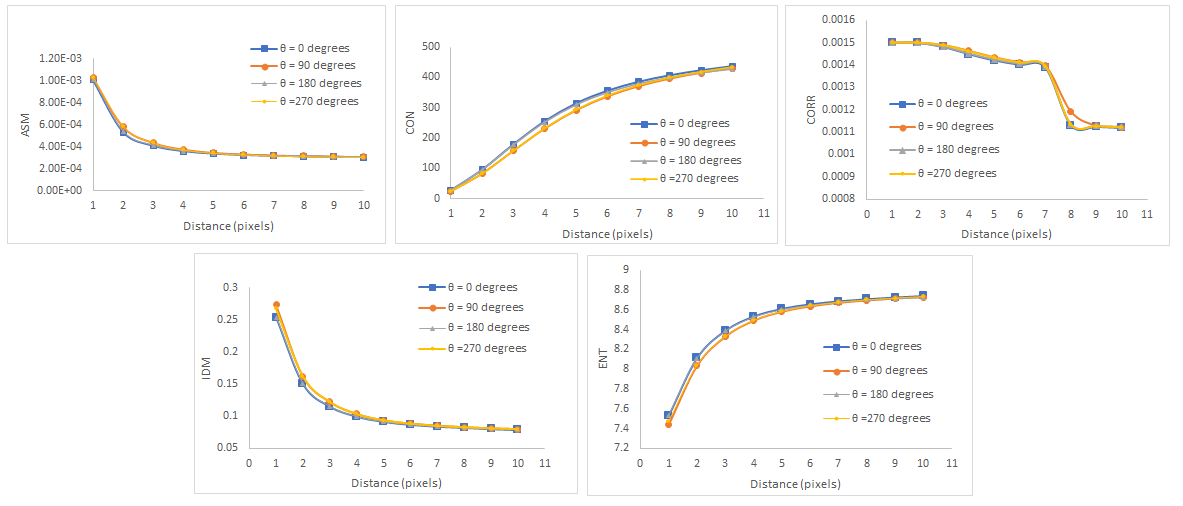
**

**Supplementary Table 1**  Binary logistic regression analysis was used to select and organize which parameters should be used in the fertilisation prediction algorithm.

**Supplementary Table 2**  Binary logistic regression analysis was used to select and organize which parameters should be used in the blastulation prediction algorithm.

**Supplementary Table 3**  Binary logistic regression analysis was used to select and organize which parameters should be used in the implantation prediction ranking tool.
